# Rebuttal to Correspondence on “DC Electric Fields Promote Biodegradation of Waterborne Naphthalene in Biofilter Systems”

**DOI:** 10.1021/acs.est.5c12499

**Published:** 2025-10-01

**Authors:** Lukas Y. Wick

**Affiliations:** Helmholtz Centre for Environmental Research - UFZ, Department of Applied Microbial Ecology, Leipzig 04318, Germany

We thank the authors for their feedback.^1^ In our article,^2^ we describe how the application of direct current (DC) electric fields to laboratory biofilter systems increased the biodegradation rate (up to 55%) of dissolved naphthalene (NAH), depending on the NAH concentration and the hydraulic flow conditions applied. We attributed the DC benefits to altered microscale flow profiles, which improve the provision of NAH to the surface-attached bacteria. DC induces electrokinetic phenomena such as electroosmotic flow (EOF) as a surface charge-induced movement of pore fluids. By acting at a nanometer distance above surfaces, it allows fluid flow to be changed where pressure-driven flow is low or inexistent. To avoid misunderstandings about our work, we here briefly address the authors’ comments.

**Kinetic Model and Numerical Data Treatment**. We acknowledge that the boundary layer model is commonly used for well-mixed fluid phases in contact with solid surfaces and that its application simplifies the complex transfer conditions in a percolation column, given its large radial and axial concentration gradients. Incorporating the model in the often-used Best equation,^3^ however, allows for an easy comparison of the overall NAH bioavailability in our system under differing experimental conditions yet does not quantitatively describe the system *sensu strictu*. In our paper, we clearly described our simplified model assumptions (reg. mass transfer coefficient (*k*) and equal bulk NAH concentrations (*C*_0_) throughout the column) and their use in the Best equation for easy comparison of different starting experimental conditions. Doing so, we calculated *k* values in a range similar to that estimated by the authors. However, by an unfortunate oversight in calculating Bn in our spreadsheet we accounted for microbial biomass in the column twice. This led to a higher Bn, which is deservedly criticized. We thank the authors for pointing this out, regret this mistake, and plan to correct it in an Addition/Correction. Although the corrected Bn values (ca. 0.007–0.035 without DC; ca. 0.008–0.131 with DC) are lower than previously reported, this does not affect the observed correlation between Bn and DC-induced biodegradation benefits and flow velocity. We acknowledge that an inflow NAH concentration of *C*_0_ can overestimate the Bn. We also derived *k* and Bn based on the bulk NAH concentration estimated by the mean of the NAH concentrations at the column inflow and outflow. While this somewhat changed *k* and Bn, it did not alter any of the described correlative trends or overall conclusions regarding the influence of DC on Bn.

**Mechanistic Interpretation Based on the EOF**. At laminar flow, the flow velocity around spherical collectors depends on the location of their surface with, for instance, low flow at the front and rear stagnation points. However, in situations where pressure and electrical drive partially counteract each other, higher dispersion or the creation of microscale turbulences (“eddies”) and likely higher NAH mass transfer and availability to cells may be expected near the bead surfaces (e.g., at the rear stagnation point). We have calculated a near surface EOF of ca. 3.4 × 10^–7^ m s^–1^. Although such an EOF is ca. 100–800 times smaller than bulk water flow in our system, the EOF allows for surface-associated liquid movement at locations where pressure driven flow approaches zero. EOF thereby may change the effective film layer thickness above surface-attached bacteria and promote the transport of NAH to the bacteria without changing the overall hydraulic flow of the bulk liquid. The EOF impact is influenced by the relative orientation of the EOF-creating surfaces to the direction of the applied electric field and the hydraulic flow. In porous materials with hydraulic flow, the EOF so can create complex flow fields that deviate from pure pressure-driven hydrodynamics by promoting inhomogeneous flow and microscale vortices in flow shadow zones.^4^ We hence propose that EOF locally changes the microscale flow profiles and NAH bioavailability, particularly in the presence of high advective flows, which is also supported by our data. We also comment that the authors, although criticizing our discussion, do not present an alternative explanation for the improved NAH biodegradation in the presence of a DC field.

**Aerobic versus Anaerobic NAH Biodegradation by *Pseudomonas fluorescens* LP6a**. The authors express concern about the reduction in the dissolved oxygen content (DO) caused by abiotic redox conditions inside the bioreactor, which could affect aerobic biodegradation. We previously excluded aerobic NAH biodegradation by measuring the DO in our systems in the presence and absence of microbial biomass without mentioning it in the article. At the electric field strength applied, we found ca. 21–28% reduced DO relative to the inflow in abiotic controls, while DO in the presence of degrading biomass depended on the biomass development. DO showed ca. 85% reduction during the exponential growth phase yet only ca. 31% reduction during the NAH biodegradation phase studied. Strain LP6a is considered an obligate aerobe, meaning that it uses oxygen as the final electron acceptor during cellular respiration and is thought to be unable to grow anaerobically. Although some *P. fluorescens* strains may utilize nitrate as an alternative electron acceptor under oxygen-restricted conditions, to the best of our knowledge, such a property has been unreported for strain LP6a. We are hence convinced that aerobic biodegradation has led to NAH removal in our system. The observed faster degradation rates in the presence of DC further would be inconsistent with the observation that biodegradation rates of organic compounds in anaerobic environments are typically lower compared than those under aerobic conditions.
